# Ultrasound guided Ru106 plaque brachytherapy for treatment of exudative retinal detachment in children with diffuse choroidal haemangioma

**DOI:** 10.1038/s41433-024-03562-8

**Published:** 2025-01-14

**Authors:** Anusha Venkataraman, Ali Al-Gilgawi, Ian Stoker, M. Ashwin Reddy, Mandeep S. Sagoo

**Affiliations:** 1 Retinoblastoma Service, Royal London Hospital, London, UK; 2 Ocular Oncology Service, Moorfields Eye Hospital, London, UK; 3 Department of Radiation Physics, St. Bartholomew’s Hospital, London, UK; 4 NIHR Biomedical Research Centre for Ophthalmology at Moorfields Eye Hospital and UCL Institute of Ophthalmology, London, UK

**Keywords:** Eye cancer, Medical imaging

## Abstract

**Purpose:**

To evaluate the efficacy of ultrasound-guided ruthenium (Ru 106) plaque brachytherapy for treatment of exudative retinal detachment in diffuse choroidal haemangioma (DCH).

**Methods:**

Retrospective analysis of four paediatric patients treated with ultrasound-guided Ru 106 plaque brachytherapy for DCH with total exudative retinal detachment directed to the thickest part of the DCH. A dose of 40 Gy to the tumour apex was delivered in all patients. The outcomes of treatment were regression of DCH, resolution of retinal detachment, development of neovascular glaucoma or any other radiation-associated complications which were assessed clinically and with B scan ultrasonography.

**Results:**

There were 4 eyes included in the study, with a mean (median, range) age of 8.75 (8.4, 3–15) years. The pre-operative tumour thickness was 5.0 (5.12, 4.2–5.5) mm. The visual acuity ranged from 0.8-2.8 LogMAR and 3 of 4 eyes had only light perception at presentation. One eye had been treated with goniotomy for pre-existing secondary glaucoma and was on topical antihypertensive medications. At a mean follow-up of 14.6 months (10.5 months, 6-30 months), all patients showed regression of the tumour. The mean tumour thickness reduced to 2.05 mm (2.44 mm, 1.1–2.6 mm) post-operatively. All patients (4/4) had complete resolution of the retinal detachment. The visual acuity remained stable in all the patients with none of the patients developing neovascular glaucoma or any other radiation-related complications.

**Conclusion:**

Ultrasound-guided Ru 106 plaque brachytherapy is an effective treatment strategy as a primary treatment in the absence of external beam radiotherapy, to achieve tumour regression and resolution of retinal detachment in DCH.

## Introduction

Diffuse choroidal haemangioma (DCH) is a vascular hamartoma frequently associated with Sturge-Weber syndrome. It usually manifests during childhood or adulthood and leads to visual loss because of hyperopia, macular oedema, amblyopia or exudative retinal detachment. It characteristically presents as a generalized orange–red choroidal thickening with a ‘tomato catsup’ appearance and it may or may not be associated with exudative retinal detachment. DCH without exudation can be generally kept under close observation. Classically, diffuse choroidal haemangiomas with exudative retinal detachments have been treated with external beam radiotherapy, but recently several studies have shown Ru 106 brachytherapy to be an effective treatment strategy.

Our study aims to evaluate intra-operative ultrasound-guided Ru106 plaque brachytherapy in the treatment of exudative retinal detachments in diffuse choroidal haemangiomas. This technique allows for more precise localization and placement of plaque adjacent to the area of maximum thickness of the haemangioma, thus achieving complete tumour regression.

## Materials and methods

This is a retrospective chart review of consecutive patients with DCH-associated retinal detachment that presented from Jan 2019 to March 2022, treated with intra-operative ultrasound-guided Ru 106 plaque brachytherapy. Informed consent was obtained from the parents of all children and approval was obtained from the Audit Department of the Royal London Hospital (Number 13107). The study adhered to tenets of the Declaration of Helsinki.

Baseline examination included best corrected visual acuity (BCVA), slit lamp biomicroscopy for neovascularization of the iris (NVI), gonioscopy for neovascularization of the angle (NVA), indirect ophthalmoscopy for the extent of retinal detachment, fluorescein angiography, and ultrasonography to determine the tumour dimensions and the area of maximum thickness of the choroidal haemangioma.

**Fig. 1:**
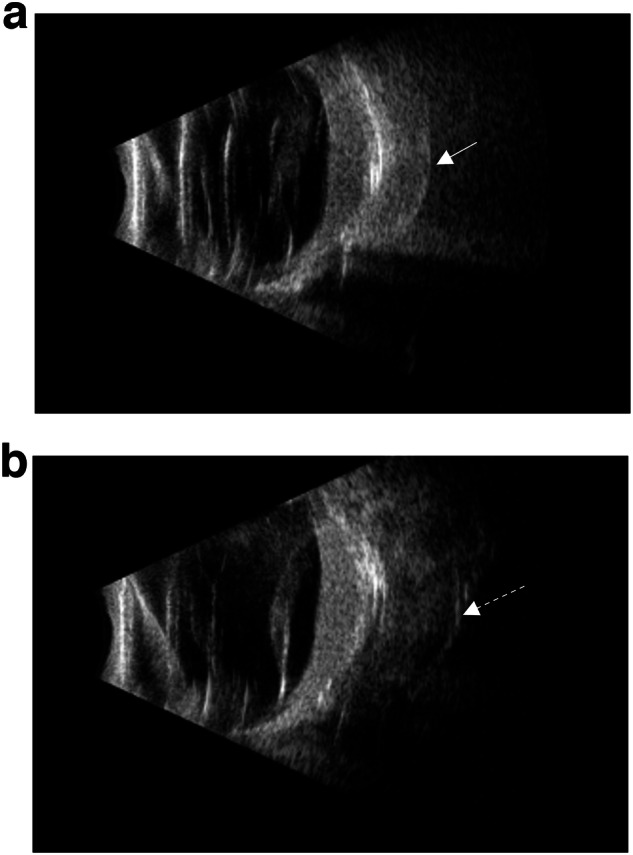
Ultrasound-guided Ru106 plaque brachytherapy for treatment of exudative retinal detachment in children with diffuse choroidal haemangioma. Images of intra-operative B scan ultrasound confirming position of dummy plaque (**a**: solid arrow) and the active plaque (**b**: interrupted arrow) adjacent to the thickest area of the tumour. (Case 3).

All patients underwent ultrasound-guided Ru 106 plaque brachytherapy with a dose of 40 Gy targeted to the tumour apex. Patients were imaged with ultrasound, and suitable patients were chosen for plaque radiotherapy. Treatment duration and rate of delivery were calculated based on the thickness of the tumour. Ultrasound was used intra-operatively to confirm the thickest part of the tumour. A 20 mm dummy plaque was initially sutured to the episcleral tissue to cover the base of the intra-ocular tumour at its thickest region. The position of the dummy plaque with respect to the tumour was checked with ultrasound (Fig. 1a). Following this, the active plaque was inserted in the place of the dummy plaque and ultrasound was used again to re-confirm the position of the plaque (Fig. 1b). The position of the plaque was accurately adjusted when found necessary. The plaque was then removed once the apex dose had been delivered. Even in older patients where EBRT could more feasibly be delivered without general anaesthetic, we chose plaque brachytherapy due to its ease of delivery, and lower complication profile, especially in treating this otherwise benign condition.

Patients were followed up at one month post-operatively, and every six months thereafter. The outcomes of treatment were regression of DCH, resolution of retinal detachment, development of neovascular glaucoma or any other radiation-related complications which were assessed clinically and with B scan Ultrasonography.

## Results

The mean (median, range) age at presentation was 8.75 (8.4, 3–15) years. The demographics and clinical details are summarized in Table 1. All were female patients. The most common presenting symptom was decreased visual acuity with the vision at presentation being light perception in three eyes and 6/36 in one eye. The choroidal haemangioma was present on the same side as the port wine stain. Exudative retinal detachment was total in 3 of 4 eyes and subtotal in 1 eye. B scan ultrasonography revealed diffuse choroidal haemangioma in all eyes with medium to high internal reflectivity. The pre-operative tumour thickness was 5 (5.1, 4.2–5.5) mm and basal diameter was 16.1 (16.8, 13.2–7.7) mm. One eye had been treated with goniotomy for pre-existing secondary glaucoma and was on topical medications.

**Table 1:** Ultrasound-guided Ru106 plaque brachytherapy for treatment of exudative retinal detachment in children with diffuse choroidal haemangioma: Demographic and clinical features.

Case	Age (years)	Gender (M/F)	Baseline BCVA	Tumour thickness (mm)	Basal diameter (mm)	Extent of retinal detachment
1.	3.2	F	PL (2.8 LogMAR)	5.04	16.3	Total
2.	15	F	PL (2.8 LogMAR)	4.24	17.2	Total
3.	3.75	F	PL (2.8 LogMAR)	5.2	17.7	Total
4.	15	F	6/36 (0.8 LogMAR)	5.5	13.2	Subtotal involving macula and Supero-temporal quadrant

Ru 106 plaques were placed as described above, with 2 eyes receiving circular 20 mm diameter plaques and 2 eyes receiving 20 mm notched plaques. Plaque parameters are summarised in Table 2. The radiation dose delivered was 40 Gy to the apex, over a mean of 49 (49, 31–67) h with the mean rate of delivery being 0.88 Gy/hr (0.82, 0.6–1.3 Gy/hr).

**Table 2:** Ultrasound-guided Ru106 plaque brachytherapy for treatment of exudative retinal detachment in children with diffuse choroidal haemangioma: Details of Ru 106 plaque brachytherapy.

Case	Type of Ru106 plaque	Radiation dose to tumour apex (Gy)	Rate of delivery (Gy/hr)	Duration of treatment (hours)	Resolution of Exudative retinal detachment	Complications
1.	CCB	40	1.29	31	100%	Nil
2.	COB	40	0.87	46	100%	Nil
3	CCB	40	0.77	52	100%	Nil
4	COB	40	0.6	67	100%	Nil

**Fig. 2:**
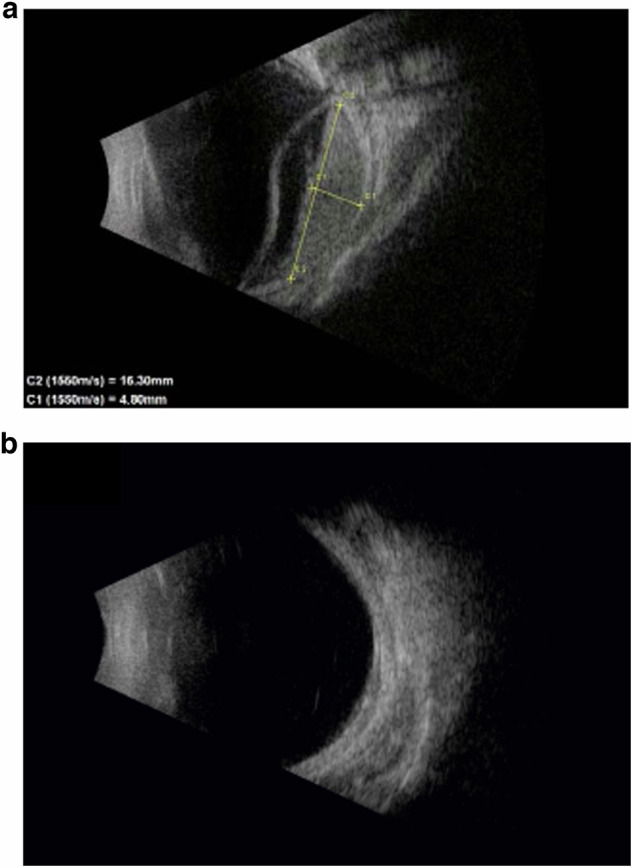
Ultrasound guided Ru 106 plaque brachytherapy for treatment of exudative retinal detachment in children with diffuse choroidal haemangioma. Pre-operative (**a**) and post-operative (**b**) B scan ultrasound images of an eye with diffuse choroidal haemangioma that underwent ultrasound-guided plaque brachytherapy showing tumour regression and resolution of exudative detachment after 7 months. (Case 1).

**Fig. 3:**
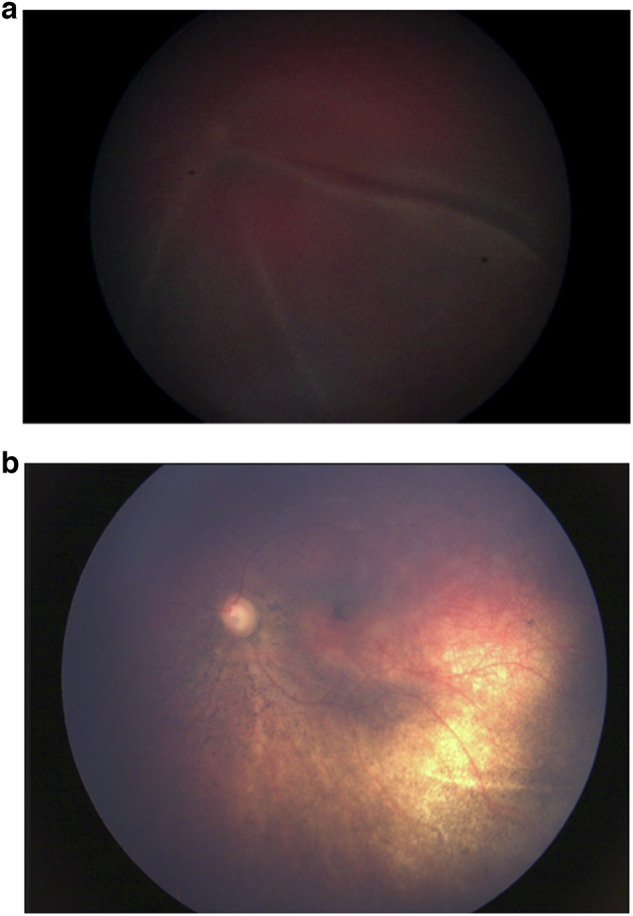
Colour fundus (Retcam) images of an eye before and after ultrasound-guided Ru 106 plaque brachytherapy for diffuse choroidal haemangioma. (Case 3). **a** Pre-operative colour fundus (Retcam) image of an eye with total retinal detachment that underwent ultrasound-guided plaque brachytherapy. **b** Post-operative colour fundus photograph image of the same eye after plaque brachytherapy showing complete tumour regression and resolution of exudative retinal detachment after 6 months.

At follow-up of 14.6 (10.5, 6–30) months following brachytherapy, all patients showed regression of the tumour with complete resolution of exudative retinal detachment (Figs. 2, 3). The tumour thickness reduced to 2.05 (2.0, 1.1–2.6) mm post-operatively. None of the patients developed recurrence, neovascular glaucoma, radiation-related complications or required secondary enucleation. Though anatomical re-attachment was observed in all the cases, the visual acuity improved only in one patient from 6/36 to 6/18 post-operatively. The other three patients retained light perception. The treatment dosing and the response to treatment have been summarized in Table 2.

## Discussion

The treatment of DCH is very challenging in the presence of total retinal detachment as it becomes difficult to visualize and thus delineate the tumour margins clinically. Multiple treatment options including laser photocoagulation, visudyne photodynamic therapy and oral propranolol therapy have been tried historically [1–5]. Each of these is suboptimal. Laser delivery to the tumour through an overlying retinal detachment often fails to provide consistent results. Treatment with oral propranolol is not effective in all cases and results in variable clinical outcomes. External beam radiotherapy has been a more successful treatment over lasers and systemic beta-blockers [6, 7]. Randon et al. showed benefit to EBRT in the treatment of DCH in Sturge-Weber syndrome but found instances of radiation-associated orbital pain, and mild cataracts in 15% of the patient population [8]. However, this was associated with damage to other ocular structures leading to cataract, radiation optic neuropathy and orbital changes leading to its abandonment in indications such as retinoblastoma [9]. It also caused a very slow resorption of subretinal fluid over several months, sometimes necessitating additional treatment for recurrence or persistence of fluid [6]. In children, external beam radiotherapy necessitates multiple general anaesthetics, carrying with that the possible risks to their long-term development [10]. Low-dose proton beam radiotherapy has been attempted successfully in two children with Sturge-Weber syndrome and extensive retinal detachments from diffuse choroidal haemangioma. The radiotherapy was delivered in 10 fractions of 2 Gy to a total of 20 Gy. One child had these fractions delivered under general anaesthetic [11].

The use of ultrasound-guided plaque therapy has been described in the management of other ocular masses, such as uveal melanoma. Tann et al. described the use of custom-made plaques to treat uveal melanoma in 48 patients who underwent ultrasound-guided plaque therapy [12]. Iodine-125 plaques were used with a reported 0% early local failure rate. Though the authors stressed the use of ultrasound guidance as pivotal in accurate application of radiation, this has not reached widespread use, as melanomas can be localised either because they block transpupillary transillumination or can be visualised by indirect ophthalmoscopy. In contrast, locating the thickest portion of a diffuse choroidal haemangioma under a bullous retinal detachment cannot use the same techniques.

There is also reported use of other imaging modalities, namely magnetic-resonance (MR), as an adjunct in plaque brachytherapy. Lewis et al. describe its use in the treatment of DCH in a young adult and suggest its use can be helpful in differentiating between tumour and subretinal fluid [13]. The authors suggested this added delineation can help to target tumours more effectively, reducing the dose required, thereby reducing the dose of radiation to normal tissue. However, performing this intra-operatively has logistical limitations.

Brachytherapy for the treatment of choroidal haemangioma was first described by Maclean et al. using radon seeds to deliver a targeted dose to the tumour without causing damage to the surrounding structures [14]. Several studies have also reported the safety and efficacy of cobalt 60, Iodine 125 and Ruthenium 106 plaques making it the treatment of choice in the current era, particularly as paediatric ocular radiotherapy facilities are becoming less common due to replacement of this modality in retinoblastoma treatment [15, 16].

Arepalli et al. reported the use of iodine-125 plaque in five eyes with DCH, four of which had total retinal detachment, one of which with subtotal [16]. They treated haemangiomas with a mean apex dose of 35 Gy and mean base dose of 76 Gy for a mean duration of 95 h. They reported complete resolution in SRF in all eyes at the seven-month follow-up. With a maximal follow-up period of 22 months, they reported no additional treatments or complications following the plaque therapy.

Kubicka-Trząska et al. described the effectiveness of Ru 106 plaque in five eyes with DCH with serous retinal detachment, though their technique of plaque placement was not stipulated [17]. They reported a target apex dose of 30.98–47.36 Gy with tumour regression and prompt resolution of subretinal fluid. However, they also found 1 out of 5 eyes developed recurrence and required repeat plaque irradiation and transpupillary thermotherapy. In contrast, none of the eyes in our study developed tumour recurrence, which may be a feature of accurate ultrasound-guided plaque placement, leading to complete tumour regression.

Similarly, Yu et al. reported Ru 106 plaque brachytherapy for DCH with serous retinal detachment, complete tumour regression was achieved with a higher median apex dose of 83 Gy (range 57–112 Gy) [18]. The plaque was directed to the thickest portion of the tumour. Of the 8 eyes, 2 required repeat treatment. However, in our study, similar results were obtained with a lower dose radiation of 40 Gy to the tumour apex with no recurrence or need for repeat treatment, by the use of intra-operative ultrasound.

In summary, this study reports the use of intra-operative ocular ultrasound to guide accurate plaque placement in patients with exudative retinal detachment associated with diffuse choroidal haemangioma. In these paediatric cases, all 4 eyes responded to the treatment with regression of the haemangioma, resolution of the retinal detachment and eye retention. However, the study is limited due to its small sample size. The poor visual outcomes are due to the late presentation with advanced retinal detachment. Treatment was offered in these cases to prevent neovascular glaucoma and a blind painful eye that would lead to enucleation.

## Conclusion

Ultrasound-guided Ru 106 plaque brachytherapy is an effective treatment strategy as a primary treatment in the absence of external beam radiotherapy, to achieve tumour regression and resolution of retinal detachment in DCH.

## Summary

### What was known before

- Plaque brachytherapy placement can be challenging in the presence of a diffuse tumour with overlying retinal detachment.

### What this study adds

- Ultrasound-guided brachytherapy is safe, efficient, and provides good results in the treatment of diffuse choroidal haemangioma especially when margins are obscured by retinal detachment.

## Supplementary information

Eye Reporting Checklist

## Data Availability

All data on ultrasound-guided plaque brachytherapy for treatment of exudative retinal detachment in children with diffuse choroidal haemangioma that support the findings of this study are included within this paper.
